# Leveraging the heterogeneity of the NK cell repertoire for the development of immunotherapies for acute leukemia

**DOI:** 10.1186/s12967-025-07093-y

**Published:** 2025-11-04

**Authors:** Enora Ferron, Maxime Jullien, Katia Gagne, Christelle Retière

**Affiliations:** 1 Etablissement Français du Sang, Nantes, 44011 Nantes, France; 2 INSERM UMR1307, CNRS UMR 6075, CRCI2NA, team 12, 44000 Nantes, France

**Keywords:** Leukemia, NK cells, Repertoire, Immunotherapies

## Abstract

Acute leukemia represents a significant therapeutic challenge, necessitating the development of innovative approaches to improve the clinical outcomes of patients. Immunotherapies have become a mainstay in the treatment of acute leukemia. Allogenic hematopoietic stem cell transplantation (allo-HSCT) is a well-established and efficacious procedure. Over time, a number of therapeutic approaches have emerged as promising strategies for achieving durable remission and have transformed the treatment of leukemia. Among the efficient immune cells engaged against leukemia, natural killer (NK) cells are innate cytotoxic cells that do not require antigenic specificity to exert their cytotoxic function. The potential of NK cells as a treatment for leukemic patients is emerging as a promising approach. They are capable of distinguishing between healthy and leukemic cells in a natural manner because of the sophisticated equilibrium between their numerous inhibitory and activating receptors. In recent years, NK cells have been found to be far more complex than expected. Indeed, the NK repertoire exhibits a remarkably high degree of phenotypic diversity, which is closely linked to the extensive polymorphism of immunogenetic KIR and HLA class I markers. It has been demonstrated that not all NK cells possess the same functional profile and that they respond to functional education mediated by these KIR-HLA molecular interactions. This review offers insights into the knowledge of NK cell diversity, with the goal of leveraging NK cell heterogeneity for the development of NK cell-based immunotherapies for acute leukemia.

## Background

Acute leukemia is a hematopoietic malignancy characterized by the uncontrolled proliferation of hematopoietic stem and progenitor cells in the bone marrow and peripheral blood, which are blocked in an immature state. These diseases are further categorized into acute myeloblastic leukemia (AML) and acute lymphoblastic leukemia (ALL), each with distinct cellular origins, characteristics, and surface markers [1, 2]. Genetically, these malignancies are characterized by high heterogeneity of mutations, contributing to their diverse clinical presentations and responses to treatment [3, 4]. The current frontline treatment of leukemia depends on various factors, primarily patient physiological age, leukemia type, and the presence of targetable molecular markers [5, 6].

In most patients, a cure is achieved only following allogeneic hematopoietic stem cell transplantation (HSCT) [7]. Despite continuous improvement in the care of patients treated with AML or ALL, resulting in improved outcomes [8–10], a significant proportion of patients still present with relapsed or refractory (R/R) diseases, which are associated with a poor survival rate. This highlights the need for the development of new therapeutic strategies.

Among these novel therapeutic modalities, chimeric antigen receptor (CAR) T-cell therapy stands out as the most transformative, significantly altering the management of relapsed/refractory B-ALL. Tisagenlecleucel is an inaugural CAR-T-cell therapy that received FDA approval in 2017 and targets the CD19 antigen on B-ALL leukemic cells [11]. Brexucabtagene autoleucel was approved in 2021 for the treatment of advanced B-cell ALL [12]. Notably, the use of anti-CD19 CAR-T cells in ALL patients has limitations, as these therapies eliminate both leukemic cells and healthy CD19⁺ B lymphocytes. To prevent immunodeficiency, patients often receive antibody replacement therapy to compensate for the loss of B cells. In patients with AML, CAR-T cells have not yet demonstrated efficacy. This is due to the absence of a specific target for AML cells, which often results in the destruction of healthy cells due to the lack of discrimination between leukemic cells and healthy cells that express the same antigen. CD33 is a well-identified target for the treatment of AML. As a myeloid differentiation antigen, CD33 is expressed on the leukemic blasts of the majority of patients with AML [13, 14], which has led to a long-standing interest in developing immunotherapies directed against CD33 for the treatment of AML. However, the expression of CD33 on normal hematopoietic progenitors restricts the applicability of anti-CD33 CAR-T cells, as their activation could result in severe and potentially lethal myeloablation.

Currently, a number of different therapies are being tested in clinical trials to ascertain their potential efficacy. As an alternative to T lymphocytes, natural killer (NK) lymphocytes represent a promising approach in the treatment of acute leukemia. In contrast to T lymphocytes, NK cells possess the intrinsic capacity to differentiate between leukemic and healthy cells. Furthermore, the NK cells employed in immunotherapy can be derived from an allogeneic donor and do not induce cytokine release syndrome (CRS) [15]. Numerous studies have underscored the phenotypic and functional heterogeneity of the NK repertoire, prompting us to consider not only the phenotypic heterogeneity of leukemic cells but also that of NK cells to optimize NK cell-based immunotherapies.

## Main text

### Natural killer (NK) cell biology

#### NK cell functions

**Fig. 1:**
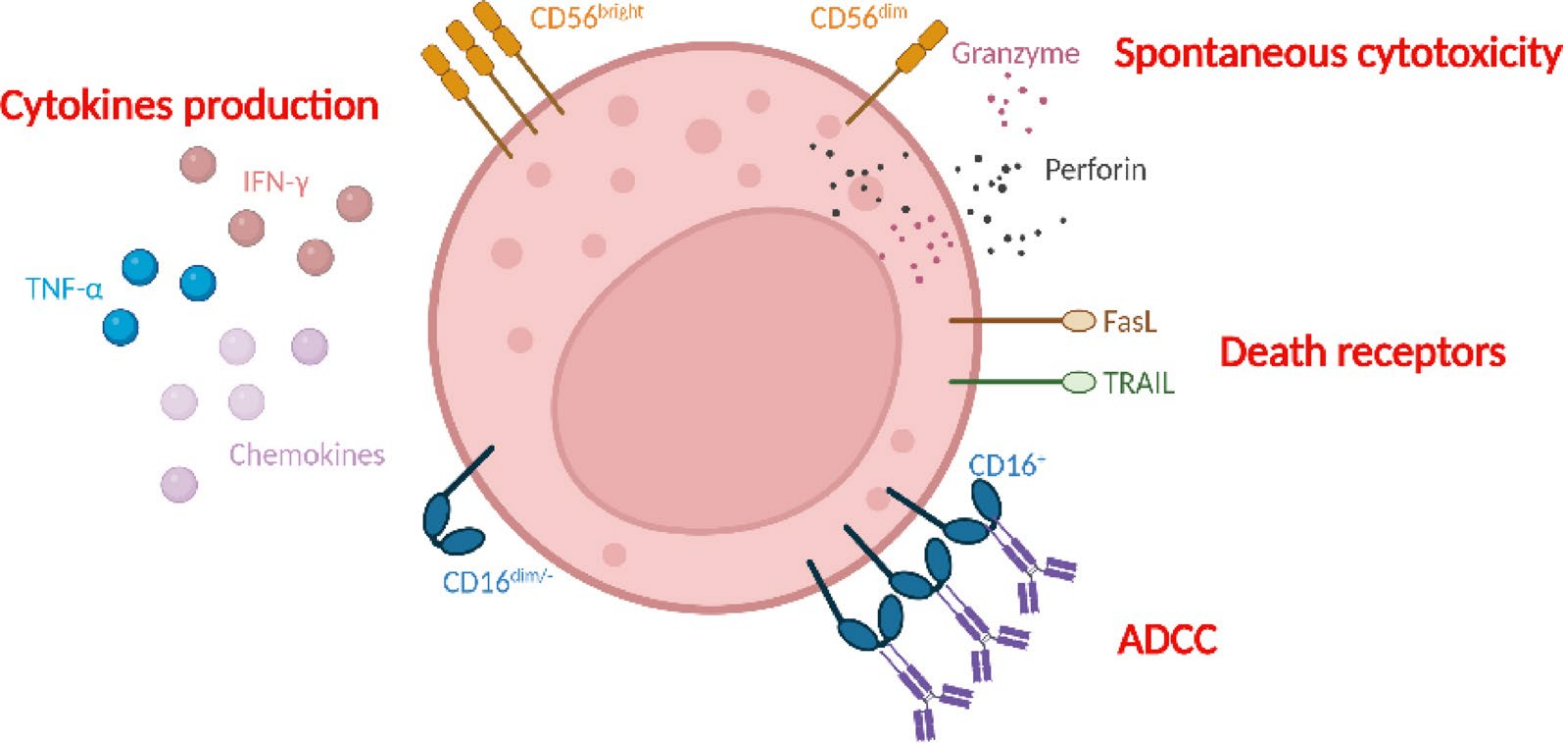
The functions of the CD56^bright^ and CD56^dim/−^ NK cell subsets. The CD56^bright^CD16^dim/−^ subset produces high levels of cytokines and chemokines but has low cytotoxic effects. In contrast, the CD56^dim/−^CD16^+^ subset is highly cytotoxic, although it displays relatively low expression of cytokines and chemokines

Natural killer (NK) cells represent a crucial component of the innate immune system, comprising 5–20% of peripheral blood mononuclear cells (PBMC) [16]. NK cells are derived from hematopoietic stem cells (HSCs), which are located in the bone marrow [17]. Once differentiated, NK cells are predominantly present in the bloodstream, although they can also be found in various lymphoid and nonlymphoid organs [18]. Human NK cells are commonly identified as CD3^−^ CD56^+^ cells. The expression of the CD56 antigen allows for the distinction of two distinct NK subsets: CD56^bright^CD16^dim/−^ and CD56^dim^CD16^+^ [19]. The CD56^dim^CD16^+^ NK cell subset is characterized by its capacity for cytotoxicity, whereas the CD56^bright^CD16^dim/−^ subset specializes in the production of various cytokines and chemokines [20, 21] (Fig. 1). NK cells exert their cytotoxicity through a number of mechanisms, including the release of lytic granules containing perforin and granzyme, leading to the destruction of target cells [20]. Additionally, NK cells can eliminate target cells by engaging the death receptors FasL and TRAIL, which bind to their receptors on the surface of target cells [22, 23]. Antibody-dependent cellular cytotoxicity (ADCC) is facilitated by the engagement of CD16a (FcγRIIIA) on the surface of NK cells, which results in the destruction of target cells [24]. NK cells also produce interferon gamma (IFN-γ) and tumor necrosis factor alpha (TNF-α), which play a role in modulating immune responses. Additionally, various chemokines are produced, which are associated with the activation of T cells [25] or the recruitment of dendritic cells [26]. Unlike T cells, NK cells are not antigen specific, which enables them to kill targets without prior immunization [27, 28]. NK cell functions arise upon cell activation, which is mediated by the engagement of activating receptors and the nonengagement of inhibitory receptors.

#### NK cell receptors

NK cells are involved in the elimination of virus-infected or cancer cells [29]. Unlike T cells, NK cells do not express somatically rearranged antigen-specific receptors. The mode of action of NK lymphocytes is based on the recognition of HLA class I molecules, which is mediated mainly by inhibitory killer cell immunoglobulin-like receptors (KIRs) and the CD94/NKGA heterodimer. The interaction of these inhibitory receptors with HLA class I molecules inhibits the functions of NK cells, which act as sentinels to ensure the expression of self via HLA molecules expressed on autologous cells. However, in a tumor context, abnormal cells can escape from T lymphocytes via mechanisms that target the reduced expression of HLA molecules [30], which are presenters of tumor antigens but then become preferred targets for NK cells. NK cells are capable of naturally recognizing the missing self via inhibitory KIRs, which do not engage with HLA molecules of the self. This principle, known as the “missing-self” theory, was described by Kärre in the 1980s [31]. Moreover, tumor cells are stressed and may exhibit aberrant expression of activating NK receptor ligands [32]. The loss of HLA class I molecule expression on leukemia cells enhances NK cytotoxicity toward leukemia targets. Taken together, fully activated NK cells require the dynamic integration of inhibitory and activating signals from a multitude of molecular receptor‒ligand interactions.

The KIR family comprises 16 genes located on chromosome 19, which can be divided into two main groups: inhibitory receptors possessing an ITIM motif (KIR2DL1, 2DL2, 2DL3, 2DL4, 2DL5, 3DL1, 3DL2, 3DL3) and activating receptors. The remaining genes, KIR2DS1, 2DS2, 2DS3, 2DS4, 2DS5, and 3DS1, possess short intracytoplasmic domains that bind to DAP12, which possesses an ITAM motif [33]. Additionally, there are nonexpressed pseudogenes, KIR2DP1 and 3DP1. KIR2DL4 is a notable exception, as it is both an inhibitory receptor and an activating receptor that binds to an adaptive protein with an ITAM motif [34]. The content of KIR genes varies among individuals, resulting in the distinction of two main KIR haplotypes (A and B) [35]. The KIR A haplotype is composed of seven functional KIR genes, with only one activating KIR (KIR2DL1, 2DL3, 2DL4, 3DL1, 3DL2, 3DL3 and 2DS4). In contrast, KIR B haplotypes are more diverse, comprising between seven and 14 functional KIR genes, with one to five B-specific KIR genes (KIR2DL2, 2DL5A, 2DL5B, 2DS1, 2DS2, 2DS3, 2DS5, 3DS1). A total of 660 KIR genotypes (AA and Bx) were identified in 25,116 individuals from 192 populations when both KIR haplotypes were considered (http://www.allelefrequencies.net/). In addition to their variable content, KIR genes are highly polymorphic, with more than 2200 KIR alleles (https://www.ebi.ac.uk/ipd/kir/). The allelic polymorphisms observed vary according to the population under study [36, 37], resulting in variable expression of KIRs on the surface of NK cells [38–40]. This polymorphism affects mainly inhibitory receptors. KIRs actively participate in NK cell functions by binding to specific amino acid sequences of HLA class I molecules, designated KIR ligands. On the basis of the amino acids at positions 77 and 80, HLA-Cw molecules are classified into two groups: C1 (Ser77 and Asn80) and C2 ligands (Asn77 and Lys80) [41]. C1 ligands are recognized by KIR2DL2/3 and KIR2DS2, whereas C2 ligands are recognized by KIR2DL1 and KIR2DS1 [42]. However, it is important to provide a nuanced perspective on this information, as KIR2DL2/3 also recognize some C2 ligands (HLA-C*05:01, C*02:02, C*04:01 allele-encoded products) and some HLA-B allotypes (B46, B73) that share similarities with C1 ligands [43, 44]. KIR3DL1 and KIR3DS1 recognize HLA-A and HLA-B molecules that contain serologic Bw4 motifs [45–49]. The Bw4 motif is distinguished by a high degree of amino acid polymorphism at positions 77 to 83 [50]. KIR3DL2 recognizes HLA-A3/A11 molecules [51, 52] as well as HLA-F [53], KIR2DL4 recognizes HLA-G molecules [54], and KIR2DS4 recognizes a number of HLA-C (C1 and C2 ligands) molecules in addition to HLA-A11 and HLA-F [53, 55]. In addition to HLA class I molecules, KIR3DL3 recognizes HHLA2 [56], whereas KIR2DL5 recognizes PVR [57]. The ligands of KIR2DS5 and KIR2DS3 are still unknown.

The NKG2 family of proteins comprises C-type lectin-like immunoreceptors. Both NKG2A and NKG2C bind to the CD94 adaptor protein. The inhibitory CD94/NKG2A complex contains an ITIM motif [58], whereas the activating CD94/NKG2C complex interacts with DAP12, which bears an ITAM motif [59]. Both proteins bind to HLA-E, which is known to present the leader sequence of classical HLA class I molecules [60]. CD94/NKG2A has a greater affinity for its ligand than does CD94/NKG2C, which results in the prevention of NK cell activation in healthy cells [61]. CD94/NKG2C^+^ NK cells or memory-like NK cells play pivotal roles in the immune response to cytomegalovirus (CMV) infection. Indeed, CMV has developed a multitude of mechanisms to evade detection and elimination by T lymphocytes and NK cells. In particular, it blocks the membrane expression of HLA class I molecules, allowing them to evade detection by T lymphocytes while simultaneously maintaining the expression of the HLA-E molecule, thereby avoiding detection by NKG2A^+^ NK cells. To achieve this, the virus produces the UL40 protein, which is similar to the signal peptide and enables stable expression of the HLA-E molecule. This expression of the HLA-E molecule induces amplification of a CD94/NKG2C^+^ NK cell subset, which coexpresses KIRs involved in NK education and the CD57 maturation marker [62–64]. In a recent study, Huisman and colleagues characterized the repertoire of human and CMV-derived peptides presented by HLA-E to CD94/NKG2A and/or CD94/NKG2C [65]. The majority of peptides can induce either inhibition or activation through CD94/NKG2A or CD94/NKG2C, respectively. However, only a limited number of peptides have been identified as capable of selectively activating CD94/NKG2C^+^ NK cells. This observation indicates that the peptide presented by HLA-E may be involved in modulating NK cell activity. The activating NKG2D receptor is a homodimer with limited homology with the NKG2 family. It can bind to the adaptor molecule DAP10 [66], which leads to the activation of NK cells. NKG2D binds to induced ligands on virus-infected or cancer cells, comprising MICA/MICB [67] and ULBP1-6 [68, 69].

The NCR family consists of three activating receptors, namely, NKp46, NKp44, and NKp30 [70–72]. They have been shown to play a role in the activation of transformed cells by NK cells. NKp46 is a hallmark of NK cells, and its binding results in the release of cytokines, cytotoxic granules and chemokines [73, 74]. The precise nature of the ligands for NCRs remains unclear, with a variety of molecules having been reported. Among the ligands, heparan sulfate proteoglycans (HSPGs), which are major components of glycoaminoglycans (GAGs), are involved in the structure of tissues [75]. HSPGs play pivotal roles in tumor progression, facilitating tumor cell proliferation, invasion of adjacent tissues and the formation of metastases [76]. In cancer cells, NKp44 is known to interact with or mixed lineage leukemia 5 (MLL5) [77], whereas NKp30 binds to B7-H6 [78]. Collectively, these receptors are important for the cytotoxicity of NK cells.

The ligands Nectin-2 and PVR are upregulated in a number of tumor cells and have been found to share multiple receptors. The activating receptor DNAX-associated molecule-1 (DNAM-1) and the immune checkpoint inhibitor T-cell immunoreceptor with Ig and ITIM domains (TIGIT) both bind to PVR and Nectin-2 [79, 80]. The inhibitory receptor T-cell-activated increased late expression (Tactile) binds exclusively to PVR [81], whereas the inhibitor PVRIG binds to Nectin-2 [82]. DNAM-1, TIGIT and Tactile bind to the same site on PVR, with TIGIT exhibiting the highest affinity, followed by Tactile and DNAM-1. To offset this, DNAM-1 is present at the NK cell surface at higher densities than TIGIT is [80]. PVRIG and DNAM-1 compete for Nectin-2, with DNAM-1 demonstrating a lower affinity [82]. It has recently been demonstrated that TIGIT also binds to Nectin-4 [83], whereas Tactile binds to Nectin-1 [84]. While DNAM-1 is associated with NK cell-mediated tumor lysis [85], TIGIT and Tactile have been shown to inhibit the cytotoxic function of NK cells [86, 87]. The precise function of the PVRIG remains uncertain. The recognition of the ligands PVR and Nectin-2 by the receptors DNAM-1, TIGIT, Tactile, and PVRIG demonstrates the intricate mechanisms regulating NK cell responses. This creates a delicate balance between the activation and inhibition of NK cells. Furthermore, the involvement of KIR2DL5, which can also bind to its own binding site on PVR, induces NK cell inhibition [88], thereby adding another layer of complexity to the regulatory mechanisms of NK cell responses.

NK cells express several inhibitory receptors that tightly regulate their activity. These include KIR, CD94/NKG2A and ILT-2, which binds to HLA class I molecules and contribute to NK cell tolerance to self. CD161 also contributes to self-tolerance as its expression is induced on NK cells following activation [89]. CD161 binds to its ligand LLT1, which is expressed on various cell types after activation [90, 91]. Beyond these canonical inhibitory receptors, emerging evidence indicates that immune checkpoint pathways also play a crucial role in NK cell dysfunction and exhaustion, particularly in the tumor microenvironment. TIGIT and CD96 have been identified as major inhibitory receptors as detailed above. Similarly, PD-1 and TIM-3 are upregulated on NK cells in the tumor microenvironment and associated with functional exhaustion [92–94]. A specific subset of PD-1⁺ NK cells have been observed in CMV^+^ individuals as well as in patients with cancer, suggesting that PD-1 expression on NK cells may contribute to NK cell dysfunction in these settings [95]. CD161 expression is induced on mature NK cells following activation and can deliver inhibitory signals [96].

NK cells express a variety of receptors that are involved in a multitude of cellular functions, including cytotoxicity, proliferation, migration and development. Indeed, NK cells express costimulatory receptors, including CD16a and 2B4. CD16a (FcγRIIIA) is a receptor that plays a pivotal role in ADCC. Upon binding to the Fc region of IgG1 and IgG3 attached to tumor cells [97], CD16a triggers the activation of NK cells, thereby enabling the destruction of these malignant cells. The activating receptor 2B4 belongs to the signaling lymphocyte activation molecule (SLAM) family and binds to CD48 [98]. 2B4 acts as an inhibitory receptor in immature NK cells that do not express the SAP adaptor molecule, which is responsible for the transduction of coactivating signals in mature NK cells [99]. NK cells also express adhesion receptors and cytokine/chemokine receptors, which are essential for NK cell functionality.

#### NK cell education

In humans, KIR2DL1/2/3 and KIR3DL1 are implicated in NK cell education through their binding to their respective ligands [100, 101]. Indeed, only NK cells that have undergone functional education to recognize KIR ligands possess the capacity for cytotoxicity, thereby enabling them to sense the absence of self on abnormal cells and eliminate them. Uneducated NK cells are present but exhibit a reduced level of responsiveness [100]. This phenomenon is referred to as licensing and allows for self-tolerance. The expression of one inhibitory receptor on the surface of NK cells likely limits the activity of NK cells [102]. Furthermore, activating KIRs modulates NK cell education and acts as a mechanism that complements education via inhibitory KIRs to secure tolerance [103]. Indeed, KIR2DS1^+^ NK cells are alloreactive against C2 targets, but only in a C1 environment [104]. In C2-positive individuals, KIR2DS1^+^ NK cells are hyporesponsive cells that maintain self-tolerance. It appears that not all KIR ligands are involved in NK cell education, as the interaction between KIR3DL2 and HLA-A3/A11 does not contribute to the education of NK cells [105]. The precise manner in which the mechanism of education is integrated and processed by NK cells remains unclear. The educational process is a continued process and may be influenced by interactions with other inhibitory or activating receptors. However, the data remain controversial with respect to education via the receptor NKG2A [106, 107]. Furthermore, the number of interactions and their intensity appear to modulate the education of NK cells [108]. Further research is needed to gain a comprehensive understanding of the educational process, which is clearly a dynamic mechanism occurring throughout the life of NK cells.

#### NK cell maturation

**Fig. 2:**
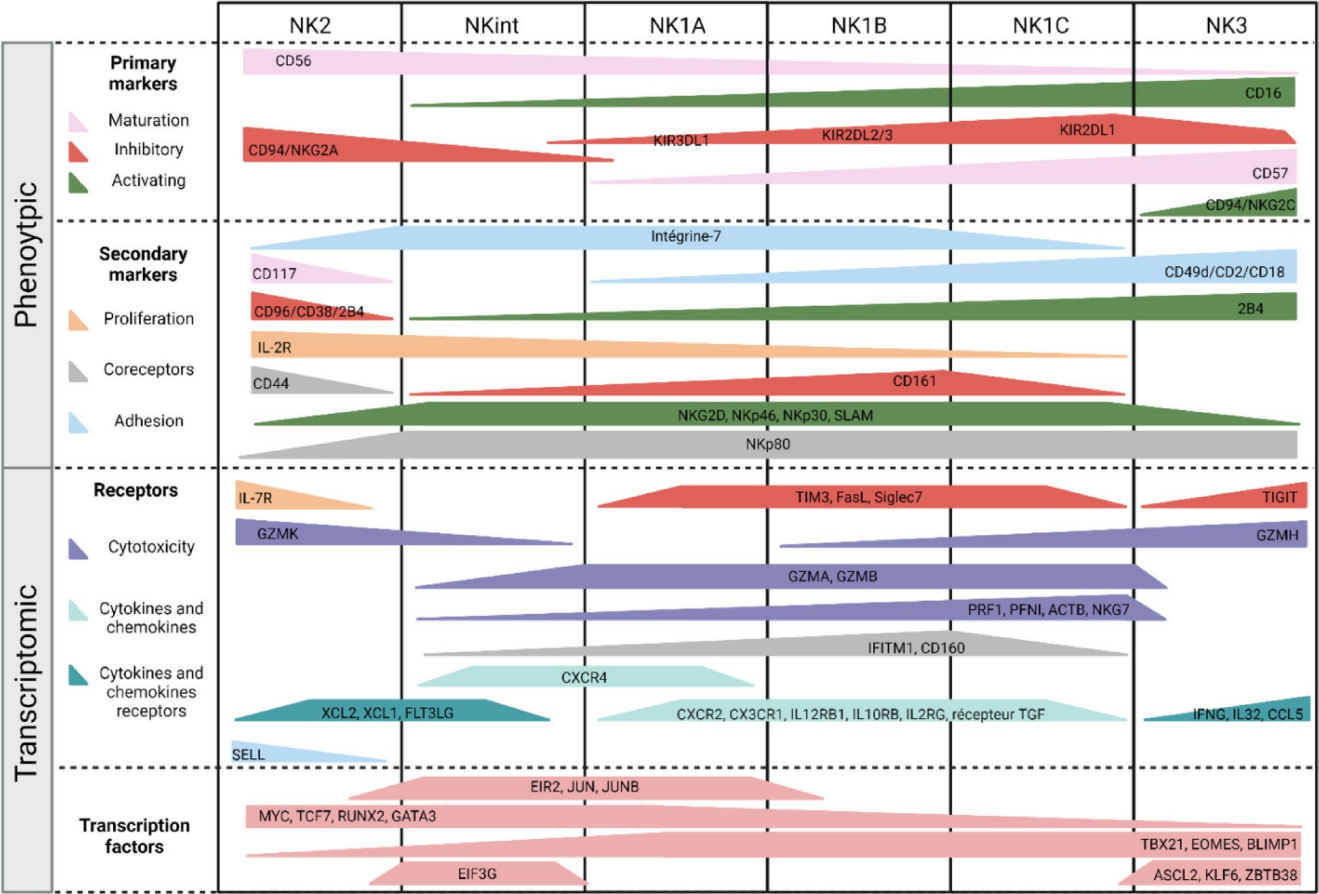
Schematic representation of NK cell differentiation. The differentiation stages of NK cells are mainly based on the CD56, CD16, NKG2A, KIR, CD57 and NKG2C receptors. Many others are involved in the phenotypic and transcriptional characterization of NK cells. The different colors represent maturation markers (light pink), inhibitory receptors (red), activating receptors (green), proliferation markers (orange), coreceptors (gray), adhesion molecules (light blue), cytotoxicity markers (purple), cytokines/chemokines (light cyan), cytokine/chemokine receptors (dark cyan) and transcription factors (dark pink)

The differentiation of NK cells is defined by the expression of specific receptors on the cell surface, which allows the identification of distinct NK cell subsets. As previously discussed, the expression of CD56 and CD16 can be used to classify NK cells into two main groups [20]. This dichotomy has also been found at the transcriptomic level by Vivier's team, with the NK1 population showing enrichment of cytotoxic signaling pathways and the NK2 population showing enrichment of cytokine production signaling pathways [109]. With the identification of new NK markers, the differentiation of NK cells has become more complex, with the characterization of several NK cell subsets within the CD56^dim^CD16^bright^ group. The model established by Björsktrom et al. [110] suggests that NK cells initially express CD94/NKG2A, followed by the expression of KIR, which seems to follow an acquisition model, with KIR2DL4 being the initial KIR expressed on all NK cells [111]. Concerning the KIRs involved in NK cell education, a study by Schönberg et al. [112] indicated that KIR3DL1 is the first acquired KIR, followed by KIR2DL2/3 and then KIR2DL1. The final step of differentiation is achieved by the acquisition of the CD57 receptor. Furthermore, CMV has been shown to influence the NK cell repertoire by inducing expansion of NKG2C^+^KIR^+^CD57^+^ subsets [62–64]. Technological advances have facilitated significant progress in our understanding of NK cell differentiation. Vivier's team performed a transcriptomic study of peripheral NK cells from healthy individuals and identified six distinct subsets that follow this differentiation pathway: NK2, NKint, NK1A, NK1B, NK1C and NK3 [113]. For each population, they defined the key transcription factors, functions, metabolic characteristics and transcriptional trajectories, allowing a more detailed description of NK cell differentiation. This study builds on and extends the results of other transcriptomic studies [114, 115]. The main features of these six NK subpopulations are described in Fig. 2.

## The phenotypic and functional heterogeneity of NK cells

NK cells can detect and selectively eliminate leukemic cells without the need for specific antigen recognition. The eradication of leukemic cells requires the formation of an immunological synapse between NK cells and target cells. Activating and inhibitory receptors are involved in binding to upregulated ligands [116] or reacting to the downregulation of inhibitory ligands [30, 117] on blast cell surfaces. The phenotypic heterogeneity of NK cells has been documented in numerous studies. A mass cytometry analysis conducted by Horowitz and colleagues [118] identified over 100,000 distinct NK cell phenotypes on the basis of the expression of 37 markers. Moreover, the stochastic expression of KIRs, in conjunction with the heterogeneous expression of activating/inhibitory receptors, gives rise to the intra- and interindividual diversity of the NK cell repertoire [35, 119]. This heterogeneity has been attributed to a number of factors, including intrinsic parameters such as genetic polymorphisms of KIR and HLA markers, variability in the expression of receptors, age and sex. As previously stated, the herpesvirus CMV, an extrinsic factor, also affects NK cell heterogeneity [62, 63, 120]. Different intrinsic and extrinsic factors influence the phenotypic organization of the NK cell repertoire [121]. In accordance with previous studies [112, 122], our findings confirm that the structure of the NK cell repertoire follows established rules and is influenced by KIR and HLA genetics. Furthermore, our findings revealed that age and sex influence the structure of the NK cell repertoire, with older age and male sex being associated with an increased frequency of mature CD57^+^ NK cells. CMV has been demonstrated to imprint the NK cell repertoire, resulting in an expansion of the memory-like NKG2C^+^KIR^+^CD57^+^ NK cell subset [61, 62]. Overall, the phenotypic organization of the NK cell repertoire is not uniform and is shaped by numerous factors throughout life. The phenotypic heterogeneity of the NK cell repertoire is associated with functional heterogeneity. NK cells exhibit a broad spectrum of responses to AML and ALL cells [123]. Notably, not all individuals are effective cytotoxic responders against leukemic cells, highlighting the existence of both intra- and interindividual functional heterogeneity among individuals. Moreover, the functionality of NK cells is also influenced by the ligands expressed on the blast surface, as ligand expression differs between ALL and AML [123, 124], resulting in distinct interactions with NK cell receptors.

## How can we leverage the heterogeneity of the NK cell repertoire for the development of immunotherapies against acute leukemia?

### NK cell-based immunotherapies for acute leukemia

**Fig. 3:**
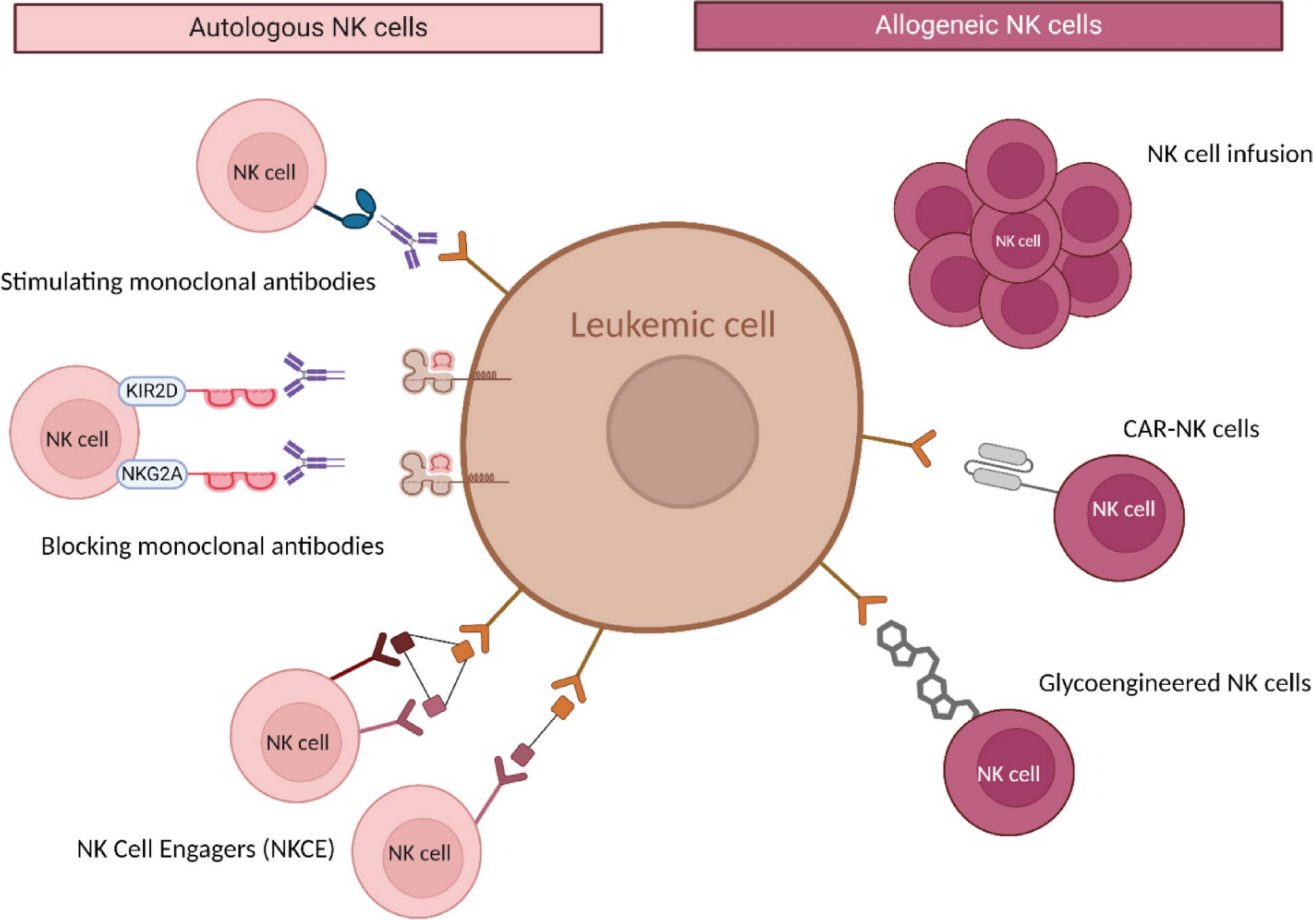
Representation of NK cell-based immunotherapies. This figure illustrates the various NK cell-based immunotherapies that are currently under development for the treatment of acute leukemia. Monoclonal antibodies can eliminate this inhibition by blocking inhibitory receptors or stimulating the patient’s own NK cells to improve NK cell ADCC function. NK cell engagers stimulate a patient’s own NK cell response. NK cell-based therapies can be derived from an allogeneic donor. These cells can be administered unmodified, through NK cell infusion, genetically modified, such as CAR-NK cells, or nongenetically modified, such as glycoengineered NK cells

A number of NK cell-based immunotherapies are currently in development for the treatment of leukemia patients (Fig. 3).

Traditional approaches use the patient’s own lymphocytes to direct the patient’s NK cells against leukemic blasts. The use of monoclonal antibodies (mAbs) represents a promising approach in NK cell-based immunotherapies. Lirilumab and monalizumab are mAbs that inhibit inhibitory KIR2DL1/2/3 and NKG2A receptors on NK cells, respectively, to improve antileukemic responses [125–128] (Table 1). Other mAbs enhance the recognition and elimination of tumor cells by NK cells through ADCC. The interaction of CD16a on NK cells with mAbs has shown significant therapeutic potential in the treatment of acute leukemia. Among these, anti-CD20 mAb, ofatumumab, was subjected to a phase 2 clinical trial (NCT01363128) involving Philadelphia^+^ ALL-B patients, during which its efficacy was proven [129]. Daratumumab monotherapy, an anti-CD38 mAb, has shown very good responses in R/R ALL patients [130, 131].

**Table 1:** NK cell-based immunotherapies currently in development for the treatment of acute leukemia

Therapy	Development stage	Pathology	Phase	Status	Trials	References
Monoclonal antibody	Monalizumab (anti-NKG2A)	AML	Phase II	Recruting	NCT06892223	[126, 127]
	Lirilumab (anti-KIR2DL1/2/3)	AML	Phase II	Complete	NCT02399917 NCT01687387	[128, 129]
	Ofatumumab (anti-CD20)	Philadelphia + ALL-B	Phase II	Complete	NCT01363128	[130]
	Daratumumab (anti-CD38)	R/R ALL	Phase II	Complete Recruting	NCT03384654 NCT05289687	[131, 132]
NKCEs	DF2001 (NKG2D, CD16, CD33) SAR’579 (NKp46, CD16, CD123)	R/R AML R/R AML, B-ALL	Phase I Phase I/II	Complete Complete	NCT04789655 NCT05086315	
	Allogeneic NK cells	AML	Pilot study	Complete	NCT00187096	[138]
Adoptive transfer	Anti-CD19 CAR-NK	ALL-B	Phase I/II	Complete	NCT03056339	[139]
	Anti-CD33 CAR-NK	AML	Phase I Phase I Phase I	Unknown Active Unknown	NCT05215015 NCT04623944 NCT05008575	

NK cell engagers (NKCEs), engineered antibody-based molecules, are currently receiving considerable attention. NKCEs such as bispecific killer cell engagers (BiKEs) and trispecific killer cell engagers (TriKEs) bring tumor and NK cells into proximity and activate NK cells, leading to leukemic cell lysis. Compared with bispecific BiKEs, TriKEs have additional antigen specificity, allowing for more precise targeting. To date, the range of NKCE options for ALL patients is limited. Preliminary in vitro studies have shown promising results for TriKEs that trigger either NKp46 or NKp30 and CD16a on NK cells, as well as CD19 on ALL cell lines and pediatric B-ALL patients [132]. In contrast, numerous NKCEs are under development for the treatment of AML patients ((Table 1), reviewed in Fenis et al. [133]). Among them, an anti-NKG2C/IL-15/anti-CD33 TriKE called NKG2C-KE that directs NKG2C^+^ cells to target CD33^+^ AML cells has shown great efficacy both in vitro and in vivo [134]. Indeed, it has been shown to induce NKG2C^+^ induced pluripotent stem cell (iPSC)-derived NK cell degranulation, IFN-γ production and proliferation of NKG2C^+^ subsets in AML cell lines and primary AML cells. Furthermore, tetravalency can increase the molecular weight of bispecific mAbs to extend their limited half-life [135].

The major limitation of the use of autologous NK cells is that patients have a deficient immune system, with reduced NK cell activity. The use of allogeneic NK cells, which are collected through apheresis from related or unrelated donor PBMCs, overcomes this major limitation. With the development of therapeutic strategies, the adoptive transfer of allogeneic NK cells has emerged as a viable approach for treating leukemic patients. NK cells can be administered via injection in an unmodified allogeneic form. The first study to investigate the allogeneic transfer of NK cells in AML patients demonstrated that the infusion of NK cells can target and eliminate AML cells, contributing to the graft-versus-leukemia (GvL) effect [136, 137]. Various sources of allogeneic NK cells have been explored, each with distinctive advantages and challenges.

Additionally, NK cells can be genetically engineered to confer specificity and enhance their cytotoxicity. The recognition of tumor antigens results in the activation of the cytotoxic capacity of CAR-NK cells, enabling the specific elimination of leukemic cells. The initial anti-CD19 CAR-NK trial in 11 patients with relapsed or refractory CD19^+^ ALL-B demonstrated no adverse effects and a favorable clinical response, suggesting the potential importance of leveraging the antileukemic properties of NK cells [15, 138]. The development of CAR therapies targeting leukemic cells in AML is significantly constrained by the lack of antigens that are exclusively expressed on AML cells. In light of the challenges associated with AML, NK cell-based immunotherapies present a promising alternative, leveraging the natural cytotoxicity of NK cells against leukemic cells while minimizing the risk of damage to healthy tissue. Despite being less developed than CAR-T cells are, CAR-NK cells engineered to target AML antigens have shown considerable promise. Anti-CD123 CAR-NK cells demonstrated high antileukemic potential with low toxicity in vitro against CD123^+^ cell lines and in vivo in two different xenograft models [139]. Currently, three clinical studies are underway to assess the efficacy of anti-CD33 CAR-NK cells in AML (NCT05215015, NCT04623944, and NCT05008575) (Table 1). A major limitation of CAR-NK therapy in AML is the lack of antigens that are exclusively expressed on leukemic cells, which increases the risk of off-target effects on healthy hematopoietic cells. Furthermore, NK cells are particularly averse to endogenous gene uptake, which results in low transgene expression [140]. Gene editing is a viable strategy to improve the antileukemic activity against primary blast cells in vitro and in vivo, for example by preventing inhibitory interactions between key NK cell receptors and their ligands on blast cells [141]. Another option is to increase NK cell persistence in vivo, either by targeting specific inhibitory pathways or by reducing host-mediated rejection [142, 143]. Fourth-generation CAR architecture incorporating IL-15 has been shown to support NK cell proliferation and persistence in vivo [15]. One challenge limiting CAR-NK efficacy is trogocytosis, a process in which NK cells acquire tumor antigens from target cells. This can lead to fratricide, where NK cells kill each other, reducing the overall therapeutic effect. To overcome this, dual CAR systems have been developed. These systems combine an activating CAR directed against the tumor antigen with an inhibitory CAR that recognizes self-antigens on NK cells, thereby protecting autologous NK cells from fratricide while preserving tumor targeting [144].

A potential alternative to NK CAR is to provide specificity only to NK cells, which are naturally cytotoxic against leukemic cells. Some research teams have developed innovative therapies, including glycoengineered NK cells. Glycoengineered NK cells are modified chemically through the insertion of glycan polymers, which attach directly to the lipids present in the cell membrane [145]. Modification of NK cell glycosylation patterns has been demonstrated to enhance the recognition of leukemic cells with altered glycosylation patterns and to improve ADCC function by optimizing binding to antibodies [146]. If different trials have validated the therapeutic potential of NK cells, the development of NK cell-based immunotherapy strategies must consider the nature of the NK cell source, the amplification protocol, and the specific target.

### The sources of NK cells

The production of CAR-T cells is restricted to the patient's own cells, whereas there is a vast range of potential sources for NK cells. The most traditional and widely studied source of allogeneic NK cells is derived from the peripheral blood of healthy donors. NK cells can be easily collected and expanded ex vivo to increase their activity and number. However, interdonor variability in the NK cell content may influence the outcome of therapy. The initial transfer of allogeneic NK cells was conducted by Miller et al. in 2005 [136]. The infusion of ex vivo expanded and activated NK cells from haploidentical donors was conducted in 19 AML patients with a poor prognosis. Prior to infusion, patients were administered a high-dose conditioning regimen comprising cyclophosphamide and fludarabine. Each patient received an infusion of 2 × 10⁷ cells/kg in addition to IL-2 injections. Despite the success of the study, complete remission (CR) was achieved in only five of the 19 patients. This was due to the conditioning and cytokine regimens, and the presence of T cells in the NK cell products was potentially sufficient to induce graft vs. host disease (GvHD). A further ten pediatric patients with AML subsequently received a purer infusion of KIR-mismatched NK cells and six doses of IL-2 [137]. Patients received a high-dose conditioning regimen comprising cyclophosphamide and fludarabine. The trial demonstrated superior outcomes, with all patients achieving remission. A number of studies have established the safety and efficacy of adoptive allogeneic NK cell therapies (reviewed in Veluchamy et al. [147]).

NK cells can be derived from HSCs found in umbilical cord blood (UCB). UCB is collected at the time of birth and stored in cord blood banks. These cells are more readily accessible and give rise to fewer ethical concerns. However, they are less mature and exhibit reduced cytotoxic potential. Moreover, their numbers are lower, and they require extensive ex vivo expansion prior to clinical use. In 2013, Knorr et al. demonstrated that human embryonic stem cell-derived NK cells can be expanded and retain their cytotoxic activity against AML and other cancer cells [148]. UCB-derived NK cells can be genetically modified further, as demonstrated by the generation of UCB-derived CD19-CAR-NK cells [149]. CAR-NK cells were obtained by transducing a CD19-CAR plasmid into IL-2- and IL-15-activated NK cells, which originated from UCB. In vitro studies of NK-CAR functionality demonstrated a high degree of degranulation against CD19^+^ ALL cells. Clinical trials of UCB-derived CAR-NK cells are ongoing in AML patients (NCT05247957 and NCT05092451).

iPSCs are reprogrammed somatic cells that can be differentiated into various cell types, including natural killer (NK) cells. iPSC-derived NK cells provide an unlimited supply of NK cells and can be genetically modified to enhance their antileukemic functions. Nevertheless, the differentiation and expansion protocols for generating fully functional NK cells from iPSCs are intricate and still undergoing optimization. Furthermore, there are safety concerns, as their potential tumorigenicity must be comprehensively addressed before their clinical application. Several models of iPSC-derived NK cells have been developed, each optimized for targeting leukemic cells. Unmodified iPSC-derived NK cells retain their natural killing ability. Indeed, unmodified iPSC-derived NK cells exhibited comparable cytotoxicity to that of peripheral blood NK cells in an in vivo ovarian cancer xenograft model [150]. The efficacy of unmodified iPSC-derived NK cells was evaluated in a clinical trial (NCT03841110) in combination with an immune checkpoint inhibitor in patients with advanced solid tumors. The administration of FT500 cells (iPSC-derived NK cells) has been demonstrated to be safe and well tolerated, with no evidence of GvHD, CRS or host immune rejection. In several studies, iPSCs have been genetically engineered to generate iPSC-derived NK cells with enhanced functions. Strategies to enhance iPSC-derived NK cell functions include the expression of secreted or membrane-bound cytokines, the knockout of specific genes to improve iPSC-derived NK cell function, the stabilization of CD16 at the cell surface or the expression of a CAR specific for a target on leukemic cells [151]. Owing to their source, NK cells do not share similar key characteristics. CD56^+^ NK cells from cord blood, HSCs and iPSCs differ in their expression of markers and cytotoxicity [152]. The main difference observed was in the KIR expression of expanded NK cells from different sources. Moreover, the authors reported that KIR^+^ NK cells displayed increased functionality in accordance with the notion that self-KIR expression enhances functionality through education [152].

NK-92 is an established NK cell line derived from a patient with lymphoma and is one of the most extensively studied NK cell lines with regard to its potential for therapeutic applications. NK92 cells are homogeneous, can be expanded to large numbers in vitro, are easily modified and have demonstrated significant antitumor activity. However, NK92 cells are derived from a malignant source and must be irradiated prior to infusion to prevent uncontrolled proliferation in patients, which significantly reduces their efficacy. NK92 cells present a characteristic phenotype in which the cell line expresses activating receptors such as NKG2D, 2B4, NKp30, and NKp46 but low levels of inhibitory receptors. In fact, the NK92 cell line expresses CD94/NKG2A and ILT-2 but not KIR (except KIR2DL4) [153]. The low expression of inhibitory receptors may explain the high cytotoxic function of these cells, which are also characterized by high levels of granzyme B and perforin [154]. This cell line can be genetically modified into CAR-NK92, which has robust antileukemic activity both in vitro and in vivo, paving the way for its use in clinical trials [155, 156]. Preliminary data from clinical trials have shown that the administration of CAR-NK92 is safe, and further trials are underway to treat leukemic patients [156].

### The amplification models of NK cells

The aforementioned sources offer distinct advantages and limitations, contingent on the clinical context and specific therapeutic goals. Moreover, there are multiple protocols for amplifying NK cells, with the objective of priming NK cells ex vivo or in vivo for optimal antileukemic functions subsequent to their infusion [157]. Indeed, freshly isolated NK cells exhibit reduced cytotoxicity in comparison with in vitro IL-2-activated NK cells [158]. The most frequently employed method of amplification is cytokine-mediated NK cell activation. To induce NK cell activation, a cocktail of cytokines, including IL-2, IL-15, IL-12 and IL-18, is used [159, 160]. IL-2 was previously administered to patients who had undergone NK cell infusion to increase NK cell persistence [136, 137]. As an alternative, ex vivo stimulation of NK cells with IL-12, IL-15 and IL-18 was proposed as a means of avoiding IL-2 infusions. However, IL-12/15/18 stimulation for up to 6 days results in significantly enhanced apoptosis and reduced cell recovery [161]. Stimulation with ALT-803, an IL-15/IL-15Rα fusion protein, results in increased NK cell cytotoxicity (perforine/granzyme and ADCC) and cytokine production in B-cell lymphoma cell lines or primary follicular lymphoma cells [162]. Cytokine-induced memory-like (CIML) NK cells differentiate following activation with IL-12, IL-15, and IL-18 and exhibit increased IFN-γ production and notable cytotoxicity against leukemic cell lines or primary human AML blasts in vitro [163]. Nevertheless, CIML NK cells function during the initial seven-day period, after which their production decreases. In vitro restimulation has been shown to restore their functions; however, further investigation is needed to elucidate the optimal methodology for restimulation. A phase I clinical trial using CIML NK cells demonstrated favorable outcomes, with clinical responses observed in five of the nine evaluable R/R AML patients, four of whom achieved CR [163]. Its efficiency has been observed in the treatment of pediatric patients with relapsed AML [164]. Recently, the inhibition of GSK3, an active serine‒threonine kinase that influences gene expression, during ex vivo NK cell expansion with IL-15 was shown to induce a more mature NK cell phenotype [165]. This phenotype is accompanied by an enhanced capacity for cytotoxicity against solid tumor cell lines.

The use of cytokines often results in limited expansion, especially when large numbers of cells are needed for therapeutic applications. To improve yield, feeder cell systems are incorporated into amplification protocols. Feeder cells provide additional stimulatory signals that mimic the natural immunological synapse between NK cells and target cells. The incorporation of feeder cells has been demonstrated to significantly increase the expansion of NK cells by providing activating ligands that engage NK cell receptors, thereby enhancing their functions. The coincubation of the CTV-1 leukemic cell line has been demonstrated to potently prime NK cells in vitro [166]. To assess the safety of this cell line, the protocol was tested in a phase I clinical trial for AML in patients at high risk of recurrence. The CTV-1 cell line was lysed, and CTV-1 lysate-primed human NK cells from a haploidentical donor were injected into 12 AML patients without exogenous cytokine support. Prior to the injection, patients received lymphodepleting fludarabine/cyclophosphamide treatment. Of the 12 patients, three exhibited durable complete remission [167]. Numerous protocols employ a variety of feeder cells, including the Epstein–Barr virus-immortalized lymphoblastic cell line (EBV-LCL), modified K562 cells or autologous PBMCs (reviewed in Maia et al. [168]). The selection of a feeder is clearly important, as amplified NK populations exhibit notable phenotypic and functional divergence depending on the feeder utilized [169].

Overall, the NK cell repertoire generated may vary depending on the source and amplification protocol. When new NK cell-based immunotherapies are designed, it is essential to consider a number of parameters. Notably, the majority of NK cell-based immunotherapies are developed from the whole repertoire with minimal consideration of NK cell heterogeneity. An understanding of NK cell heterogeneity should facilitate the definition of the source and amplification model of NK cells. Notably, AML and ALL blasts, which originate from distinct cellular lineages and exhibit unique surface marker profiles, are recognized differently by NK cells. A comprehensive understanding of the main molecular interactions between NK cells and both AML and ALL is essential for advancing our knowledge in this field.

### Different molecular interactions between NK and leukemia cells

**Fig. 4:**
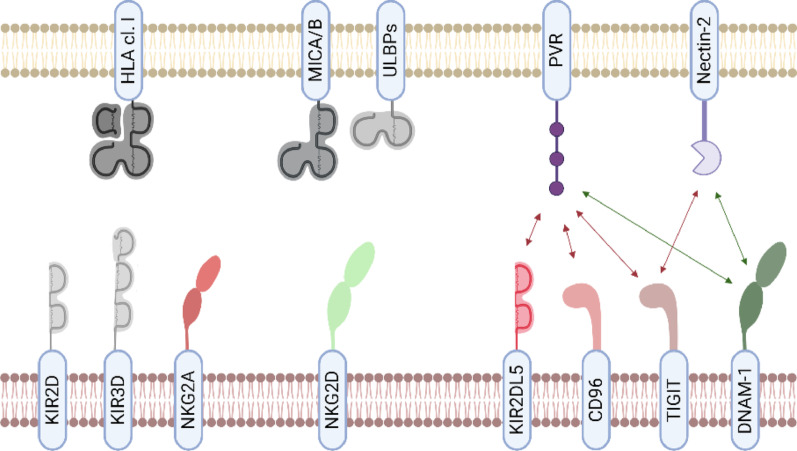
Overview of the main molecular interactions between NK receptors and ligands on leukemic cells. Leukemic cells express ligands that bind to NK cell receptors. Among these ligands, HLA class I molecules that bind to KIRs and CD94/NKG2A are downregulated, particularly in AML cells. Conversely, AML cells overexpress PVR, a ligand for the inhibitory receptors KIR2DL5, CD96, and TIGIT and the activating receptor DNAM-1. MICA/MICB and ULBPs, NKG2D ligands, and nectin-2, ligands of TIGIT and DNAM-1, are upregulated in both ALL and AML cells

The differential expression of ligands of NK cell receptors may be responsible for the observed variation in the efficiency of each NK cell subset against leukemic cells. As multiple receptors are involved in the lysis of acute leukemic cells, determining which ligands of NK receptors are expressed by ALL and AML cells is highly important (Fig. 4).

Studies have shown that ALL cells express higher levels of HLA class I molecules than AML cells do [123, 170, 171]. This observation provides insights into the mechanism by which KIR^+^ NK cell subsets exert their antileukemic effects. The absence of interactions between inhibitory KIRs and HLA class I molecules results in the triggering of NK cell cytotoxicity. Numerous studies have been conducted on the expression of upregulated ligands on leukemic cells, with some yielding conflicting results. ALL cells express low levels of PVR, whereas AML cells express high levels of PVR [171–173]. The expression of Nectin-2, ULBPs and MICA/MICB is more disparate on the surface of ALL and AML, with differences apparent according to the type of pathology and age [170–172]. Nevertheless, these ligands may be more abundant on the surface of AML cells. Recently, Kaito et al. demonstrated that high expression of PVR and Nectin-2 in AML cells is associated with a poor prognosis [174]. DNAM-1 seems to play an important role in the recognition of leukemic cells through its binding to Nectin-2. The role of DNAM-1 was subsequently corroborated by another study, even though elimination occurred by binding to PVR [175]. NKG2D appears to play a significant role in the immune response against AML cells, as the absence of NKG2D ligands is associated with a poor prognosis for AML patients. [176]. Further research is needed to gain a deeper understanding of the ligands expressed on the ALL and AML cell surfaces. Taken together, these studies highlight the potential of leveraging specific NK cell subsets for therapeutic interventions.

### Potential avenues to improve NK cell-based immunotherapies

Patients’ own NK cells have reduced NK cell activity. Leukemic cells can evade NK cell-mediated immune responses through various mechanisms, inducing phenotypic alterations and NK cell hyporesponsiveness. Indeed, several studies have demonstrated reduced expression of activating NK receptors [177, 178], defaults in adhesion molecules [179], and expansion of hyporesponsive NK cell subsets [180]. In contrast, allogeneic NK cells display efficient responses, yet their intra- and interdiversity imply that there are both good and bad donors and good and bad NK cell subsets with strong cytotoxic profiles [123, 171]. Many researchers have dedicated efforts to identifying the best donor or the most suitable NK cell subset for the development of NK cell-based immunotherapies [105, 175, 181, 182]. Some of these focus on CMV^+^ blood superdonors who harbor large preexisting memory-like single KIR^+^NKG2C^+^ NK cell subsets that exhibit efficient cytotoxicity against HLA class I-mismatched AML [183]. Rezvani’s team has observed the varying characteristics of UCB, with a particular focus on how the phenotypic differences of the infused NK cells determine the clinical outcome [138]. Notably, UCB-derived NK cells coexpressing the activating receptors NKG2D, CD16 and 2B4 appear to have a highly functional phenotype.

In light of the current understanding of the heterogeneity of the NK cell repertoire and the nature of the molecular interactions between NK cells and leukemic cells, potential avenues for enhancing the efficacy of NK cell-based immunotherapies can be proposed. The efficacy of NK cell-based immunotherapies could be enhanced by a greater understanding of the specific NK cell populations responsible for the eradication of ALL and AML cells. Depending on the source of the NK cells and the amplification protocol, the phenotypic profile of the most effective NK cells will differ. Among resting PBMCs, NKG2A^+^KIR^−^ NK cells appear to be the most efficient NK subset against ALL cells [123, 184]. In contrast, AML cells are most effectively eliminated by alloreactive KIR^+^ NK cells in the context of T-cell-depleted haplo-HSCT [185]. Specifically, the NKG2A^+^KIR2DL3^+^ and NKG2A^+^KIR2DL1^+^ NK cell subsets (referred to as NKG2A^+^KIR^+^) represent the most effective NK cells against AML [171]. In many approaches, NK cell purity is achieved by magnetic CD3 depletion and/or CD56 selection. In this way, it is conceivable to improve NK cell selection by focusing on the most effective NK cell subsets.

**Fig. 5:**
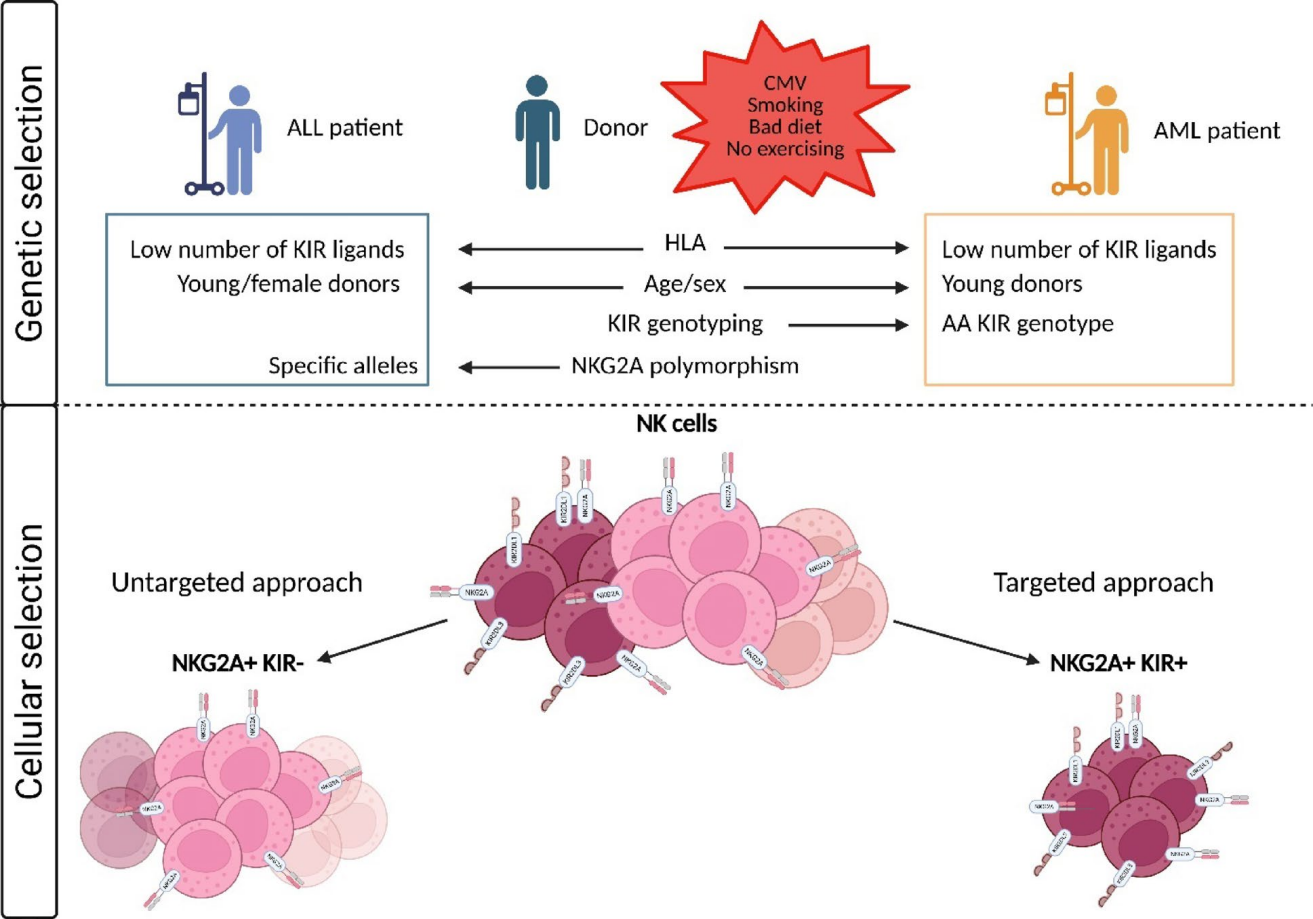
Suggested model for the optimization of NK cell-based immunotherapies. For ALL patients, an untargeted approach seems to be the most appropriate approach, given that the NKG2A^+^KIR^−^ subset is the most prevalent subset in the NK cell repertoire. In the case of AML patients, a targeted approach should be appropriate given that the NKG2A^+^KIR^+^ cell subset is less represented in the NK cell repertoire. To ensure optimal representation of each subset, the donor should be selected on the basis of the number of KIR ligands and the NKG2A polymorphism for ALL patients or the KIR AA genotype for AML patients

NKG2A^+^ NK cells from healthy blood donors have been identified as a particularly effective subset against ALL cells and constitute the predominant cell type within the NK cell repertoire [118, 121]. The functionality of these cells can be attributed to the phenotypic expression of multiple activating receptors, such as NKp30, NKp46, 2B4 and NKG2D, and to the transcriptomic expression of *GZMK*, which encodes granzyme K [113, 171]. Accordingly, a nontargeted approach utilizing total NK cells from a donor should be appropriate (Fig. 5). Furthermore, this NK cell subset displays high proliferative capacity, as demonstrated via an in vitro model of IL-15 NK cell stimulation [186]. However, the source and amplification approach should maintain the intrinsic phenotypic and functional characteristics of this NK cell subset. Indeed, UCB-derived NK cells constitute a large proportion of NKG2A^+^ NK cells, but they display an immature phenotype [187]. The NK92 cell line may be a suitable option for ALL patients, as these cells express CD94/NKG2A but not KIR [153]. However, their malignant profile should impact their phenotypic and functional characteristics, which should differ from those of the healthy donor NKG2A^+^ NK cell subset.

It appears that younger and female donors are the most suitable candidates for exhibiting a higher frequency of the NKG2A^+^ NK cell subset. Moreover, a reduction in KIR ligand diversity and the absence of the HLA-A3/A11 ligand have been reported to result in a greater proportion of NKG2A^+^ cells [102] [121]. Another criterion that has been identified as influencing the proportion of NKG2A^+^ NK cells is the allelic polymorphism of NKG2A [188]. Specifically, some alleles have been shown to improve the frequency of this subset within a patient, resulting in a diminished NK KIR^+^ subset. Taken together, these intrinsic parameters could optimize the selection of the NK cell donor.

As in ALL patients, consideration of the heterogeneity of the NK repertoire would further enhance the development and efficacy of NK cell-based immunotherapies for AML. A recent study reported that six AML patients who had relapsed following haplo-HSCT were treated with an infusion of a specific CIML subset after receiving lymphodepleting chemotherapy [189]. In addition, patients received seven doses of IL-2 to increase expansion and improve the persistence of NK cells. CIML infusion resulted in rapid in vivo expansion that was sustained for many months, with clinical responses observed in four of the six patients. However, although this NK subset has attractive properties, it has been shown that it is not the most effective NK subset against AML cells [123, 171, 190]. Resting NKG2A^+^KIR^+^ NK cells constitute the most effective NK cell subset against AML cells. The functionality of these cells can be attributed to the phenotypic expression of the activating receptors NKp30, DNAM-1, 2B4 and NKG2D and to the transcriptomic expression of cytotoxic molecules such as *GZMA*, *GZMB*, *PRFI*, *PFNI*, *ACTB* and *NKG7* [113, 171]. However, their low prevalence within the overall NK cell repertoire suggests their selection and expansion to optimize NK cell-based immunotherapies against AML (Fig. 5). Furthermore, the amplification process should be facilitated by the high proliferative capacity of this NK cell subset, as previously demonstrated [186]. Donors may be selected on the basis of HLA class I and KIR genotypes. On the basis of HLA-A, HLA-B, and HLA-C typing, the identification of donors that possess both C1 and C2 ligands and are Bw4 positive will facilitate the acquisition of an important pool of educated KIR^+^ NK cells, which will provide the most efficient antileukemic effect. On the basis of KIR typing, the selection of a donor with an AA genotype, which is known to encompass the most highly expressed and efficient KIR2DL1, KIR2DL3 and KIR3DL1 allotypes, will result in the acquisition of beneficial NK cell alloreactivity [39, 191, 192]. Indeed, the KIR2DL1*003 and KIR2DL3*001 allele-encoded products, which are characteristic of AA genotypes such as KIR3DL1^high^ allotypes, are highly expressed on NK cells, thereby facilitating the superior education and function of NK cells. Moreover, as the interaction of KIR2DL5 with PVR has been demonstrated to inhibit NK cell functions [88] and PVR is highly expressed on the surface of AML cells, selecting a donor with a KIR AA genotype seems to be a more effective strategy for the elimination of AML cells [88, 171]. The protocol for amplification of NKG2A^+^KIR^+^ NK cells should be fine-tuned to the behavior of KIR^+^ NK cells in culture. Indeed, a cocktail of the cytokines IL-12/15/18, as opposed to IL-15 alone, has been shown to drastically limit KIR2DL2/3 and KIR2DL1 expression on the NK cell membrane after 36 h [161]. Moreover, IL-12/15/18-activated NK cells are less sensitive to KIR2DL2/3-mediated inhibition. In contrast, the protocol based on HLA class I-negative B-cell lines used as feeders considerably increases the frequency of KIR^+^ NK cells that do not recognize their cognate ligands on feeder cells [104, 193].

More generally, the CD57 marker appears to be linked to age, as blood donors over 50 years of age present a more differentiated CD57^+^ NK cell repertoire than do blood donors under 50 years of age [121]. Avoiding the selection of resting CD57^+^ NK cells for therapeutic application seems to be an important criterion. Indeed, these cells are often considered exhausted and exhibit reduced cytotoxic functionality [123, 171, 194]. This may explain why several studies have reported that these methods are ineffective in various diseases [123, 195]. Moreover, CD57^+^ NK cell subsets display a lower proliferation capacity than their CD57^−^ counterparts do [186]. Notably, after in vitro stimulation, new CD57^+^ NK cell subsets that do not share the same characteristics as resting CD57^+^ NK cells emerge. The memory-like NK cell subset is associated with CMV infection and expresses CD57. Although its functional potential is lower than that of NKG2A^+^KIR^−/+^ cell subsets in acute leukemia, the memory-like NK cell subset has a greater functional capacity in CMV^+^ individuals than in CMV^−^ individuals [171], as previously reported [175].

Taken together, these approaches warrant investigation as potential avenues for improving patient outcomes. The selection of an appropriate NK cell donor for the treatment of acute leukemia should consider additional parameters beyond those related to HLA and KIR genotyping or the educational state of NK cells. Indeed, additional factors may influence the efficacy of NK cell-based immunotherapies, including specific donor age, sex, CMV status, and additional NK cell receptors, which may influence treatment outcomes. Other parameters, such as smoking, exercise, or diet, have been shown to impact NK cell biology and may therefore be considered for donor selection [196]. Gaining a greater understanding of all the parameters that drive the functional NK cell repertoire will be essential in developing new strategies for profiling NK cells on the basis of functional potential.

## Conclusions

In conclusion, the diversity of the NK cell repertoire is identified as a critical parameter in the development of new therapeutic strategies for the treatment of acute leukemia. The enhancement of NK cell-based immunotherapies for the treatment of acute leukemia requires a comprehensive understanding of the specific NK cell subsets and their interactions with leukemic cells. The process of donor selection should be refined by integrating genetic and cellular-based donor selection models, considering both KIR and HLA class I genotypes and the functional state of NK cells. Furthermore, additional factors, such as donor age, sex, CMV status, and the presence of inhibitory receptors on NK cells, must be considered to optimize the efficacy of NK cell-based immunotherapies. A comprehensive approach that incorporates these parameters will enhance the therapeutic potential of NK cell-based immunotherapies, offering more effective and personalized immunotherapeutic interventions and allowing improved outcomes for patients with acute leukemia.

## Data Availability

Not applicable.

## Abbreviations

ALL: Acute lymphoblastic leukemia
AML: Acute myeloblastic leukemia
ADCC: Antibody-dependent cellular cytotoxicity
BiKEs: Bispecific killer cell engagers
CAR: Chimeric antigen receptor
CR: Complete remission
CIML: Cytokine-induced memory-like
CMV: Cytomegalovirus
DNAM-1: DNAX-associated molecule-1
GAGs: Glycoaminoglycans
GvHD: Graft vs host disease
GvL: Graft versus leukemia
HSCs: Hematopoietic stem cells
HSCT: Hematopoietic stem cell transplantation
HSPGs: Heparan sulfate proteoglycans
iPSCs: Induced pluripotent stem cells
IFN-γ: Interferon gamma
KIRs: Killer cell immunoglobulin-like receptors
MLL5: Mixed lineage leukemia 5
mAbs: Monoclonal antibodies
NK: Natural killer
NKCEs: NK cell engagers
PBMC: Peripheral blood mononuclear cells
R/R: Relapsed or refractory
SLAM: Signaling lymphocyte activation molecule
Tactile: T-cell activation increased late expression
TriKEs: Trispecific killer cell engagers
TNF-α: Tumor necrosis factor alpha
UCB: Umbilical cord blood
